# Complete mitochondrial genome of the snakehead (*Channa gachua*) and its phylogeny

**DOI:** 10.1080/23802359.2019.1693930

**Published:** 2019-11-20

**Authors:** Bin He, Xingguo Liu, Yingying Huan, Xuan Che, Tao Yan, Jungang Yan, Zhihai Long, Bin Li, Zheng-Yong Wen

**Affiliations:** a The Fishery Institute of the Sichuan Academy of Agricultural Sciences, Yibin, China;; b Fishery Machinery and Instrument Research Institute, Chinese Academy of Fishery Sciences, Shanghai, China;; c College of Life sciences, Conservation and Utilization of Fishes resources in the Upper Reaches of the Yangtze River Key Laboratory of Sichuan Province, Neijiang Normal University, Neijiang, China

**Keywords:** *Channa gachua*, mitochondrial genome, phylogenetic analyses

## Abstract

In present study, the mitochondrial genome (mitogenome) of *Channa gachua* was determined and the phylogenetic relationship of Channidae fish was reconsidered. The mitogenome of the *C. gachua* is 16547 bp in length, containing 13 protein coding genes (PCGs), 22 transfer RNA genes (tRNAs), two ribosome RNA genes (rRNAs), a control region (D-loop) and an origin region of replication on the light-strand (O_L_). The overall nucleotide composition is 28.32% A, 26.58% T, 29.41% C, 15.69% G, with 54.90% AT, respectively. Phylogenetic analyses revealed that *C. gachua* belongs to the genus *Channa* and shares a close relationship with *C. marulius* and *C. striata*.

*Channa gachua* (Hamilton 1822), is a small and colorful fish species of family Channidae (Jearranaiprepame 2017), it is widely distributed in Asia, and now it has been listed as an endangered species in Singapore and China (Paray et al. 2017). Thus far, most researches related to this species are mainly focused on nutritional value calculation (Hariati et al. 2019), environmental indication (Ali et al. 2019), morphological identification (Jearranaiprepame 2017) and pathogenic biology (Chaudhary et al. 2019). However, the phylogenetic status of this species is still unclear due to its limited genetic information.

Here, we determined the mitogenome of *C. gachua* using the NGS sequencing technology. Specimens were collected from Jinghong city in Yunnan province of China (100.80°E, 22.01°N), and a juvenile (Te-Cg2017-01) was deposited in the Herbarium of Neijiang Normal University. The mitogenome of *C. gachua* (GenBank: MF924390) is 16547 bp in length, which is similar to those of the other Channid fishes. The overall nucleotide composition is 28.32% A, 26.58% T, 29.41% C, 15.69% G, with 54.90% AT, which is consistent with that in *C. asiatica*, *C. maculate* and *C. striata* (Wang et al. 2013; Meng and Zhang 2016). Meanwhile, the circular molecule possesses a typical piscine mitochondrial order comprising 13 PCGs, 2 rRNAs, 22 tRNAs, a D-loop and an O_L_ regions, suggesting the structures of fish mitogenome are highly conserved (Wen et al. 2017; Li R et al. 2019).

Phylogenetic analyses were performed using Maximum likelihood (ML) and Bayesian analysis (BI) methods based on two concatenated datasets of 13 PCGs at both nucleotide and amino acid levels. The tree was clustered into two groups of family Channidae and other families, and the Channidae group was further divided into two clades of genus *Channa* and genus *Parachanna* (Figure 1). Similar results also can be found in several previous molecular and morphological studies (Benziger et al. 2011; Little et al. 2012), which further confirms that the family Channidae comprises of genera *Channa* and *Parachanna*. In addition, both trees showed that the *C. gachua*, *C. striata* and *C. marulius* were grouped in one clade (Figure 1), suggesting their close relationship. These findings will undoubtedly provide useful genetic information about *C. gachua*, which also can be selected as a new genetic marker for studying population genetics.

**Figure 1.**
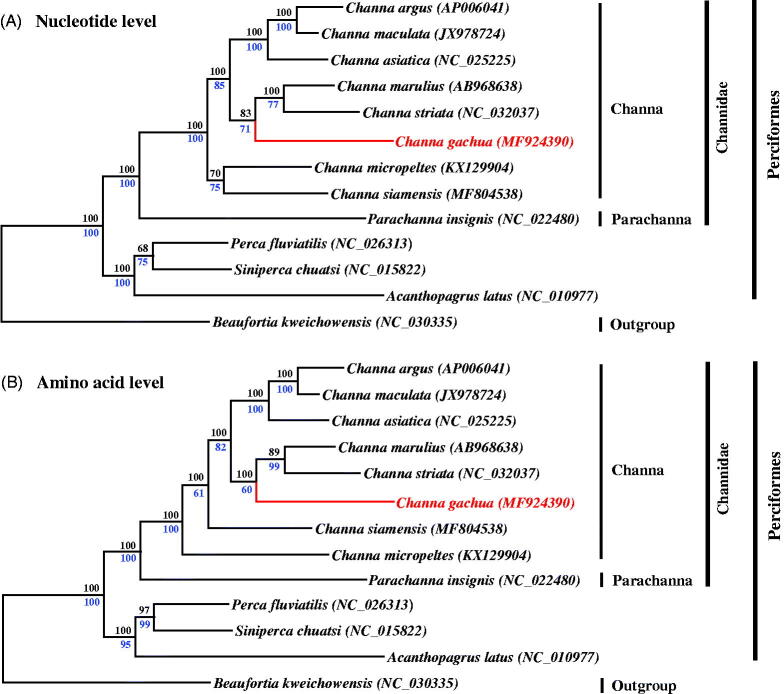
Phylogenetic analyses infer the relationship among Channid fishes. Two methods including maximum likelihood (ML) and Bayesian inferences (BI) were used to reconstruct the trees at both nucleotide (A) and amino acid (B) levels. The tree topologies produced by ML and BI analyses were equivalent. Bayesian posterior probability (blue number) with 2,000,000 generations and bootstrap support values for ML analyses (black number) with 1000 replicates are shown on the nodes. The *C. gachua* is shown in red type. GenBank accession numbers of mitogenomes are presented after the taxon name. *Beaufortia kweichowensis* (Wen et al. 2017) was selected as an outgroup.
